# Carotid napkin-ring sign plaques are associated with early neurological deterioration in lenticulostriate artery branch atheromatous disease

**DOI:** 10.3389/fneur.2026.1822751

**Published:** 2026-08-10

**Authors:** Rui Huang, Man Qu, Qiao Lin, Qiuyue Chen, Linkao Chen, Taotao Tao, Jiaolei Jin, Xinwei He

**Affiliations:** Department of Neurology, Taizhou Central Hospital (Taizhou University Hospital), School of Medicine, Taizhou University, Zhejiang, China

**Keywords:** branch atheromatous disease, carotid artery, early neurological deterioration, napkin-ring sign, stroke

## Abstract

**Background:**

Early neurological deterioration (END) is a frequent and devastating clinical feature of branch atheromatous disease (BAD). The napkin-ring sign (NRS) on computed tomography angiography (CTA) has emerged as a surrogate imaging marker of vulnerable atherosclerotic plaques. This study aimed to investigate the association between carotid NRS plaque burden and END in patients with lenticulostriate artery (LSA)-territory BAD.

**Methods:**

We retrospectively enrolled 247 consecutive patients with LSA-territory BAD. Patients were categorized into END and non-END groups. Carotid NRS plaque presence and area were assessed using CTA. Intracranial arterial stenosis (ICAS) in non-culprit vessels was also evaluated. Associations between carotid NRS plaques, END, and ICAS were analyzed using multivariable logistic regression and receiver operating characteristic (ROC) curve analyses.

**Results:**

END occurred in 25.9% of patients. The prevalence and area of carotid NRS plaques were significantly higher in patients with END than in those without END, both ipsilaterally and contralaterally (all *p* < 0.05). These associations remained significant after adjustment for age, sex, and baseline NIHSS score. ROC analysis demonstrated moderate predictive performance of NRS plaque area for END (AUC = 0.778 ipsilateral; AUC = 0.692 contralateral). Furthermore, patients with ICAS exhibited a significantly higher prevalence of carotid NRS plaques.

**Conclusion:**

Increased carotid NRS plaque burden is independently associated with END and ICAS in patients with LSA-territory BAD. Carotid NRS plaques may serve as a potential imaging biomarker for identifying BAD patients at high risk of neurological deterioration.

## Introduction

Branch atheromatous disease (BAD) is a distinct subtype of ischemic stroke characterized by subcortical infarction within the territory of a penetrating artery in the absence of significant stenosis of the parent large artery. It accounts for approximately 10–20% of acute ischemic strokes and is reported more frequently in Asian populations (1, 2). Pathologically, BAD is thought to result from atherosclerotic plaque extension at the origin of the perforating artery or occlusion of the proximal segment of the perforator by parent artery plaque (1, 2).

A notable clinical feature of BAD is its tendency toward early neurological deterioration (END), particularly progressive motor weakness (1–3). END has been consistently associated with worse functional outcomes and remains difficult to predict at an early stage. Although several studies have examined parent artery plaque characteristics and vascular geometry in relation to END, reliable imaging markers that can be readily obtained in routine clinical practice are still lacking (3–5). Identifying such markers is important, as timely recognition of high-risk patients may influence therapeutic strategies.

The napkin-ring sign (NRS), first described in coronary computed tomography angiography (CTA), refers to a low-attenuation core surrounded by a higher-attenuation rim (6, 7). Emerging evidence suggests that NRS serves as a potential imaging biomarker for high-risk plaque features (6, 7). NRS plaques could improve risk stratification for adverse events and help predict future major adverse cardiovascular events (6, 7). The presence of plaques with NRS indicates advanced atherosclerotic lesions with high specificity (3, 8). The value and clinical relevance of NRS plaques on the carotid artery remain unclear. Previous studies have identified carotid NRS plaques on cervicocerebral CTA as an imaging marker of vulnerable plaques, and have shown that they are associated with an increased risk of ischemic stroke and cerebral small vessel disease (6, 9).

Among penetrating artery territories, the lenticulostriate artery (LSA) region is particularly vulnerable to BAD-related progression because of its complex anatomical course and limited collateral supply (10). Considering that atherosclerosis is a systemic process, carotid plaque vulnerability may reflect a broader pro-atherogenic or inflammatory milieu that also affects intracranial vessels. We therefore hypothesize that carotid NRS plaque burden may be associated with END in patients with LSA-territory BAD.

In this study, we aimed to investigate the association between carotid NRS plaque burden and early neurological deterioration in patients with LSA-territory BAD. We further explored the relationship between carotid NRS plaques and intracranial arterial stenosis in non-culprit vessels.

## Materials and methods

### Patient enrollment

This retrospective study included consecutive patients with LSA-territory BAD who were admitted to the Department of Neurology at Taizhou Central Hospital (Taizhou University Hospital) between January 2020 and December 2023.

Inclusion criteria were: (1) age between 18 and 80 years; (2) admission within 48 h of symptom onset; and (3) diagnosis of LSA-territory BAD based on imaging findings, defined as: (a) diffusion-weighted imaging (DWI)-confirmed acute infarction in the LSA territory extending over ≥ 3 consecutive axial slices (slice thickness 5–7 mm); and (b) < 50% stenosis in the ipsilateral parent artery supplying the perforator, including the middle cerebral artery, internal carotid artery, or common carotid artery.

Exclusion criteria were: (1) presence of other intracranial lesions at baseline (e.g., hemorrhage, vascular malformation, aneurysm, abscess, malignant tumor); (2) cardioembolic stroke etiology; (3) stroke due to other determined causes; (4) prior carotid endarterectomy, carotid stenting, or intracranial thrombectomy; (5) incomplete clinical data; or (6) poor imaging quality.

Two experienced neurologists independently reviewed all imaging data to confirm the diagnosis of BAD according to lesion morphology and vascular territory. Discrepancies were resolved by consensus.

This study was approved by the Ethics Committee of Taizhou Central Hospital [Approval No. 2025-5-15]. Written informed consent for participation was not required for this study, in accordance with national legislation and institutional requirements.

### Baseline data collection and definitions

Demographic characteristics, vascular risk factors (including hypertension, diabetes mellitus, and dyslipidemia), smoking and alcohol consumption status, and medication history were extracted from electronic medical records. Definitions of vascular risk factors were consistent with previously published criteria (9, 11, 12).

**Figure 1:**
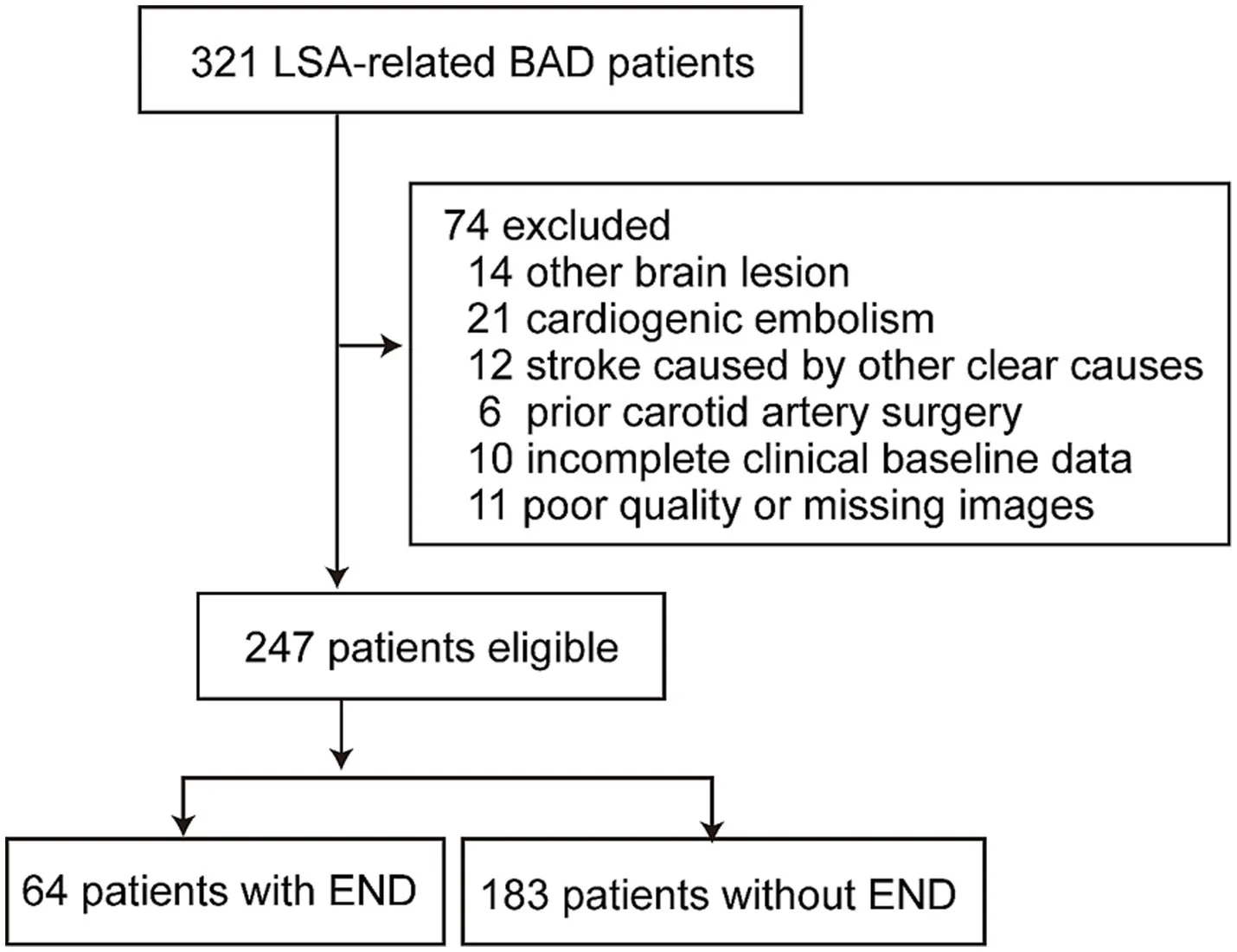
Flow diagram of patient screening and inclusion. LSA, lenticulostriate artery; BAD, branch atheromatous disease; END, early neurological deterioration.

END was defined as an increase of ≥ 2 points in the total National Institutes of Health Stroke Scale (NIHSS) score or ≥ 1 point in the motor item score compared with the best neurological status within the first 7 days after symptom onset (10, 13). END attributable to secondary causes—such as symptomatic hemorrhagic transformation, metabolic disturbances, medication-related adverse effects, or severe systemic complications—was excluded Figure 1.

Infarct volume was manually measured on DWI axial images using ImageJ software. The total infarct volume was calculated by multiplying the lesion area of each slice by slice thickness.

Intracranial arteries were evaluated using CTA, magnetic resonance angiography, or digital subtraction angiography. Moderate-to-severe ICAS was defined as ≥ 50% luminal narrowing in non-culprit intracranial vessels according to established criteria in previous studies (14).

### Neuroimaging acquisition and processing

CTA examinations were performed using a 64-slice CT scanner (Discovery CT750 HD, GE Healthcare, United States). Standardized acquisition parameters included 100 kVp, 3 mAs, slice thickness of 0.625 mm, and a field of view of 250 × 250 mm. Ioversol contrast medium (1.5–2 mL/kg) was administered intravenously at 4.0 mL/s using a power injector.

**Figure 2:**
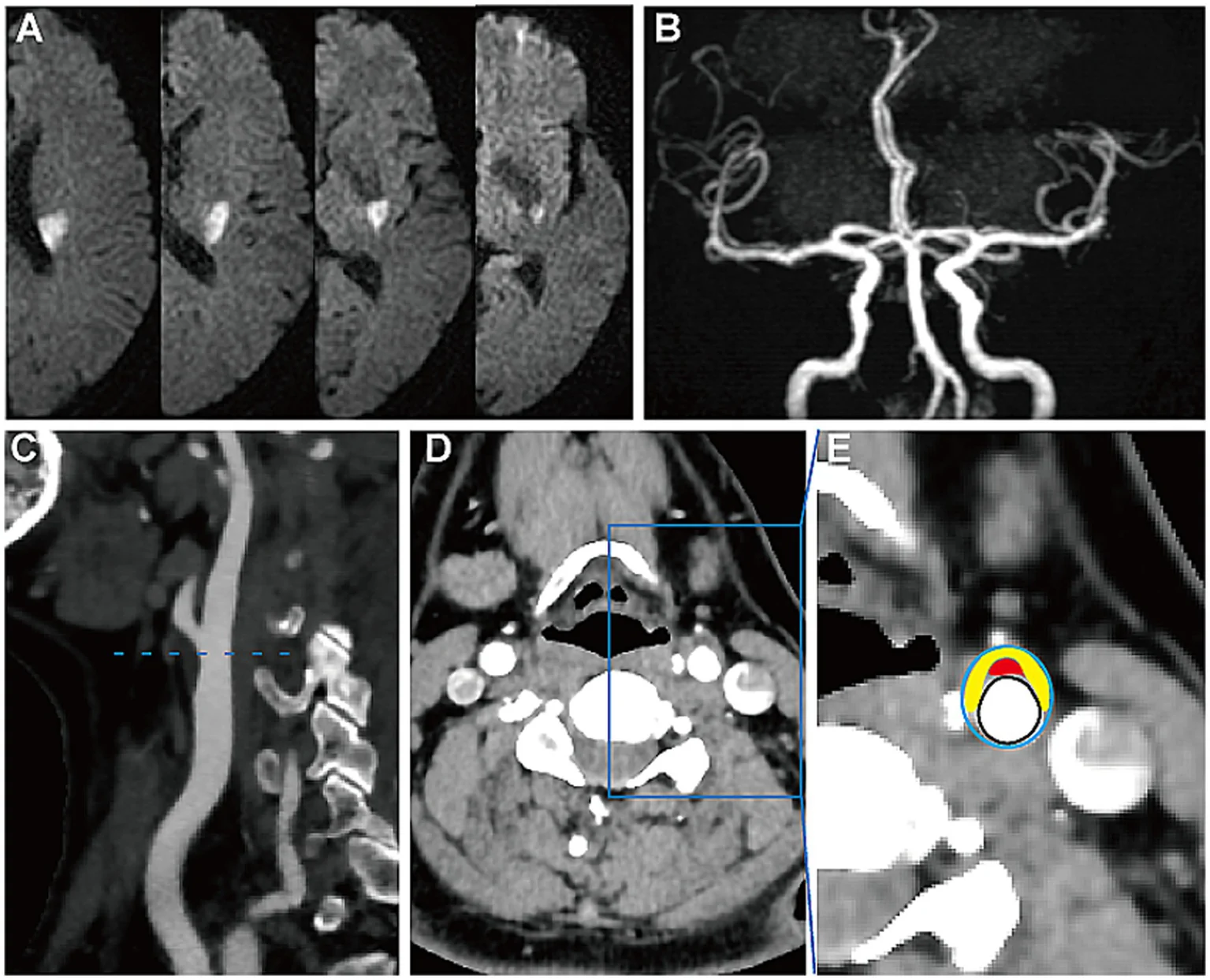
Representative imaging findings in a patient with LSA-territory BAD. **(A)** Diffusion-weighted imaging demonstrates an acute infarction within the LSA territory. **(B)** Magnetic resonance angiography shows no significant stenosis in the ipsilateral parent artery of the responsible vessel. **(C)** The reconstructed image reveals mild stenosis of the Left Common carotid artery. **(D,E)** Computed tomography angiography identifies a NRS plaque in the Left Common crotid artery. The low-attenuation core (red) is surrounded by a higher-attenuation rim (yellow), while the contrast-enhanced lumen appears white. LSA, lenticulostriate artery; BAD, branch atheromatous disease; NRS, napkin-ring sign.

The NRS on CTA has been defined in previous studies as follows: an inner low-density core surrounded by an outer high-density ring ≤ 130 Hounsfield units (9, 15). Previous studies have identified the carotid artery as one of the most common anatomical sites for NRS plaques (6, 9). In this study, we selected axial CTA images spanning from the bilateral common carotid arteries to the origin of the internal carotid arteries (representative example is shown in Figure 2).

Quantitative measurement of NRS plaques was performed using ImageJ software (version 1.54f, National Institutes of Health, United States). Two blinded raters manually segmented the full boundary of each NRS lesion on axial CTA images in strict accordance with the standardized definition of the napkin-ring sign. For carotid arteries harboring multiple separate NRS plaques, only the maximum cross-sectional area of the largest lesion was recorded for subsequent statistical analyses. Any disagreements regarding plaque identification and area measurement were resolved via joint discussion with a third senior neuroradiologist specializing in cerebrovascular imaging to reach a consensus.

### Statistical analysis

Normally distributed continuous variables are expressed as mean ± standard deviation (SD), and non-normal continuous variables are expressed as median [interquartile range (IQR)]. Categorical data are presented as frequencies (percentages). Comparisons of continuous variables between two groups were performed using Student’s *t*-test, paired *t*-test, analysis of variance with Bonferroni correction, Mann–Whitney U test or Pearson’s chi-square test as appropriate. Inter-rater reliability for bilateral NRS plaque area measurement was assessed using intraclass correlation coefficients (ICC). Multivariate analyses were performed using logistic regression models adjusted for potential influencing factors. We constructed two multivariable logistic regression models. Model 1 was adjusted for age and sex (mandatory demographic variables), together with baseline NIHSS score and infarct volume (variables with significant between-group differences that strongly predict neurological deterioration). We then conducted a sensitivity analysis with Model 2, in which traditional vascular risk factors were added into Model 1 to test the stability of the primary association. All data were analyzed using SPSS 20.0 (IBM, Chicago, IL, United States). Two-sided *p* < 0.05 were considered statistically significant if not otherwise specified.

## Results

### Characteristics of the patients

A total of 247 LSA-territory BAD patients were included. Patients were divided into END and non-END groups, including 64 (25.9%) patients with END and 183 (74.1%) patients without END.

There were no significant between-group differences in age, sex distribution, or conventional vascular risk factors, including hypertension, diabetes mellitus, dyslipidemia, coronary heart disease, smoking status, and alcohol consumption. However, patients who developed END presented with significantly higher baseline NIHSS scores (*p* < 0.001), higher infarct lesion volume (*p* = 0.005) and a higher prevalence of ICAS (*p* < 0.001) compared with those who remained neurologically stable (Table 1).

**Table 1:** Comparisons between patients with or without END.

Variables	END *N* = 64	No-END *N* = 183	*P*
Age, years	67.6 ± 11.3	66.7 ± 12.4	0.616
Male, *n* (%)	43 (67.2%)	108 (59.0%)	0.248
Hypertension	54 (84.4%)	145 (79.2%)	0.371
Diabetes mellitus	34 (53.1%)	74 (40.4%)	0.078
Dyslipidemia	36 (56.3%)	114 (62.3%)	0.394
Coronary heart disease	9 (14.1%)	26 (14.2%)	0.977
Smoking	25 (39.1%)	62 (33.9%)	0.455
Drinking	12 (18.8%)	46 (25.1%)	0.300
Admission NIHSS	5 (2, 7)	3 (1, 5)	<0.001
Intravenous thrombolysis	9 (14.1%)	23 (12.6%)	0.759
Antiplatelet therapy			0.429
Mono antiplatelet therapy	8 (12.5%)	36 (19.7%)	
Dual antiplatelet therapy	17 (26.6%)	43 (23.5%)	
Other	39 (60.9%)	104 (56.8%)	
Stenosis of intracranial artery			<0.001
None or Mild (<50%)	32 (50%)	141 (77.0%)	
Moderate (50–69%)	21 (32.8%)	36 (19.7%)	
Severe (≧70%)	11 (17.2%)	6 (3.3%)	
Infarct lesion volume (cm^3^)	4.2 ± 0.6	3.9 ± 0.6	0.005

The values are presented as the mean ± SD or median (interquartile range) for continuous variables and as a number (percentages) for categorical variables. NIHSS, National Institutes of Health Stroke Scale; END, early neurological deterioration.

### Association between carotid NRS plaques and END

Inter-rater agreement was quantified using ICC, demonstrating excellent reliability: 0.855 (*p* < 0.001) for right-sided measurements and 0.868 (*p* < 0.001) for left-sided measurements.

**Figure 3:**
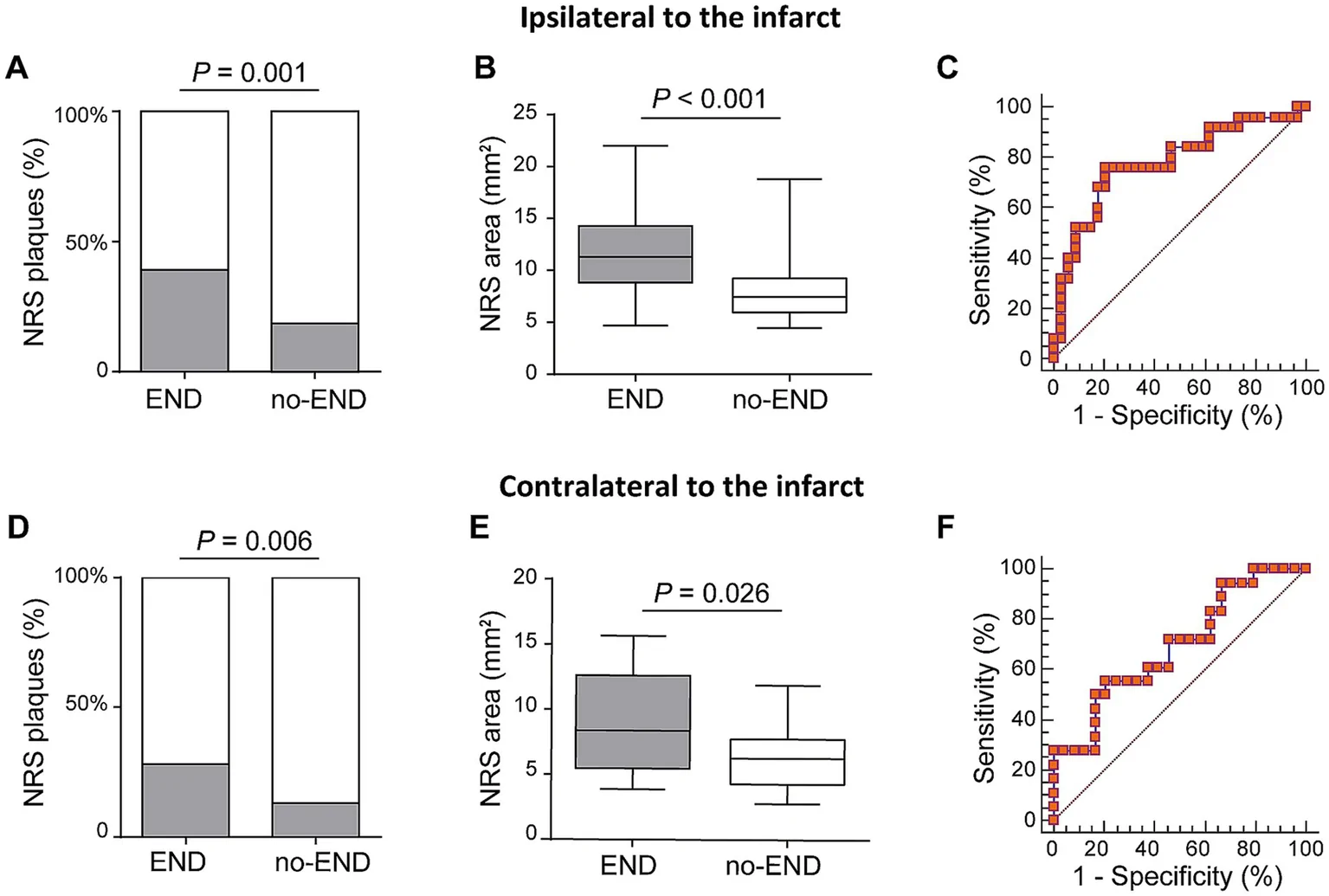
Association between carotid NRS plaques and END in patients with BAD. **(A–C)** Comparisons between patients with and without END on the ipsilateral side. **(D–F)** Comparisons between patients with and without END on the contralateral side. NRS, napkin-ring sign; END, early neurological deterioration; BAD, branch atheromatous disease.

NRS plaques were detected in 23.9% of patients on the ipsilateral side and 17.0% on the contralateral side. The prevalence of NRS plaques was significantly higher in patients with END than in those without END, both ipsilaterally (39.1% vs. 18.6%, *p* = 0.001; Figure 3A) and contralaterally (28.1% vs. 13.1%, *p* = 0.006; Figure 3D). We constructed two multivariable logistic regression models to verify the independent association between carotid NRS plaques and END. In Model 1 (adjusted for age, sex, baseline NIHSS score and infarct volume), the presence of NRS plaques remained independently associated with END on both the ipsilateral side (OR = 2.484, 95% CI 1.309–4.714, *p* = 0.005) and contralateral side (OR = 2.598, 95% CI 1.252–5.392, *p* = 0.010; Table 2). To test the robustness of our findings, we further built Model 2 with additional adjustment for conventional vascular risk factors including hypertension, diabetes mellitus, dyslipidemia, smoking and alcohol consumption. The significant associations persisted in Model 2 (ipsilateral NRS plaques: OR = 2.672, 95% CI 1.378–5.184, *p* = 0.004; contralateral NRS plaques: OR = 3.000, 95% CI 1.405–6.405, *p* = 0.005; Table 2).

**Table 2:** Association of NRS plaques and END after adjusted to clinical parameters.

Characteristics		Ipsilateral side		Contralateral side	
		OR (95% CI)	*P*	OR (95% CI)	*P*
Model 1	NRS plaques	2.484 (1.309, 4.714)	0.005	2.598 (1.252, 5.392)	0.010
	NRS area	1.284 (1.056, 1.561)	0.012	1.366 (1.053, 1.773)	0.019
Model 2	NRS plaques	2.672 (1.378, 5.184)	0.004	3.000 (1.405, 6.405)	0.005
	NRS area	1.338 (1.067, 1.677)	0.012	1.333 (1.013, 1.753)	0.040

Model 1, each parameter was adjusted for age, sex, infarct lesion volume and NIHSS at baseline. Model 2, each parameter was adjusted for age, sex, infarct lesion volume, NIHSS at baseline, and conventional vascular risk factors (hypertension, diabetes mellitus, dyslipidemia, smoking and drinking). NRS, napkin-ring sign; END, early neurological deterioration; NIHSS, National Institutes of Health Stroke Scale.

For NRS plaque area, patients with END exhibited larger NRS lesions than patients without END, both ipsilaterally [12.3 (9.6, 15.4) mm^2^ vs. 7.1 (5.0, 9.8) mm^2^, *p* < 0.001, Figure 3B] and contralaterally [8.1 (5.8, 11.9) mm^2^ vs. 4.2 (3.8, 6.9) mm^2^, *p* = 0.026, Figure 3E]. In Model 1, larger NRS plaque area was independently linked to END on both sides (ipsilateral: OR = 1.284, 95% CI 1.056–1.561, *p* = 0.012; contralateral: OR = 1.366, 95% CI 1.053–1.773, *p* = 0.019; Table 2). After full adjustment in Model 2, the correlation remained statistically significant (ipsilateral NRS area: OR = 1.338, 95% CI 1.067–1.677, p = 0.012; contralateral NRS area: OR = 1.333, 95% CI 1.013–1.753, *p* = 0.040; Table 2).

Receiver operating characteristic (ROC) curve analysis of the NRS area for END showed that the area under the curve (AUC) values were 0.778 (95% CI, 0.651–0.876, *p* < 0.001, Figure 3C) with cut-off value of 9.3 mm^2^ on symptomatic side, and 0.692 (95% CI, 0.531–0.825, *p* < 0.001, Figure 3F) with cut-off value of 7.8 mm^2^ on the contralateral side.

### Association between carotid NRS plaques and ICAS

**Figure 4:**
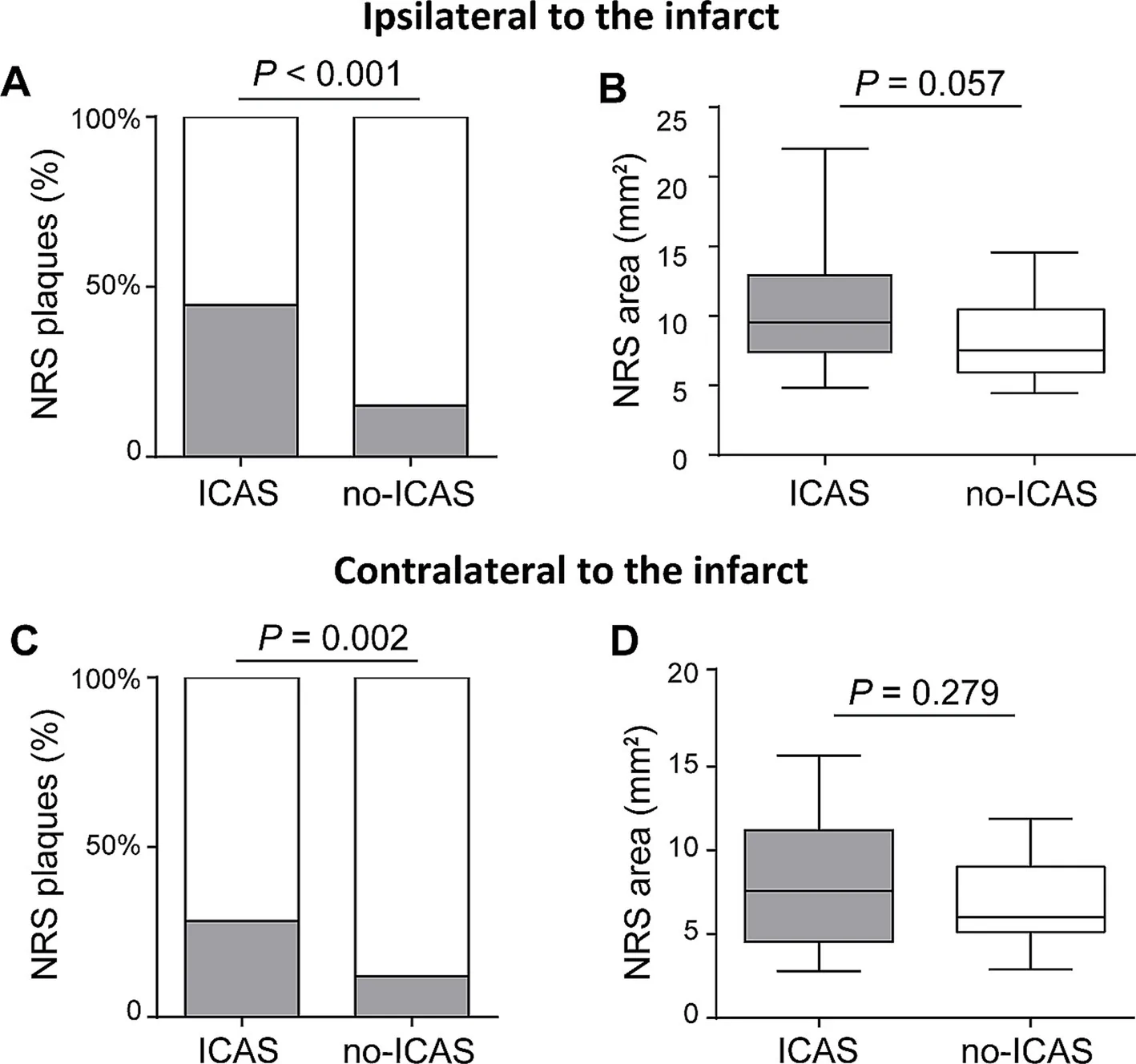
Association between carotid NRS plaques and ICAS in patients with BAD. **(A,B)** Comparisons between patients with and without ICAS on the ipsilateral side. **(C,D)** Comparisons between patients with and without ICAS on the contralateral side. NRS, napkin-ring sign; ICAS, intracranial arterial stenosis; BAD, branch atheromatous disease.

Moderate-to-severe ICAS (≥50% luminal stenosis) in non-culprit vessels was identified in 74 patients (30.0%). Patients with ICAS had a significantly higher prevalence of NRS plaques than those without ICAS, both on the ipsilateral side (44.6% vs. 15.0%, *p* < 0.001, Figure 4A) and on the contralateral side (28.4% vs. 12.1%, *p* = 0.002, Figure 4C).

For NRS plaque area, no difference was found between patients with or without ICAS, whether ipsilaterally [10.2 (9.2, 14.0) mm^2^ vs. 5.0 (5.0, 13) mm^2^, *p* = 0.057, Figure 4B], or contralaterally [7.1 (3.8, 9.5) vs. 4.7 (4.2, 6.9), *p* = 0.279, Figure 4D].

## Discussion

In this study, we found that carotid NRS plaque burden was associated with END and moderate-to-severe ICAS in patients with LSA-territory BAD. Both the presence and the area of NRS plaques were higher in patients who developed END, and these associations persisted after adjustment for potential confounders.

Recent studies have demonstrated that BAD-related stroke exhibits a higher prevalence in Asian populations, with a marked predisposition for END and significantly poorer outcomes (13). The mechanisms underlying END in BAD are likely multifactorial. Proposed explanations include local thrombosis superimposed on an ostial atheroma, thrombus propagation along the perforating artery, and instability of the parent artery plaque (8). Previous studies using whole-brain vessel wall imaging have further suggested that plaques located proximally to the origin of the lenticulostriate artery may independently predict END in acute BAD (3, 8).

CTA is widely used in the evaluation of cerebrovascular disease and allows visualization of plaque characteristics beyond simple luminal stenosis (16). Previous study found that carotid siphon calcification and its attenuation values may predict symptomatic progression in BAD (17). Our observations further indicated that pathological features frequently extend beyond the parent arterial wall, suggesting a complex interplay between focal plaque pathology and systemic vascular vulnerability.

In recent years, growing research efforts have been focused on identifying the characteristic markers of atherosclerotic plaque vulnerability (18, 19). Previous cardiovascular studies have shown that NRS in coronary plaques has been identified as pathologically thin-cap fibroatheromas with high rupture susceptibility (19). Extrapolating from coronary artery evidence, ring-like enhancement on imaging reflects intensive vasa vasorum neovascularization, a histopathologically validated feature of advanced atherosclerotic lesions that strongly associated with plaque vulnerability (18, 20). The necrotic core triggers macrophage activation, inducing secretion of pro-inflammatory cytokines (21). The synergistic interplay between neovascularization and inflammation constitutes the primary pathophysiological mechanism underlying plaque vulnerability and subsequent rupture (20, 21). In addition, the specificity of the NRS to identify advanced lesions is excellent in coronary atherosclerotic plaques (15).

In the present study, patients with END exhibited larger NRS plaque areas compared with those without END. This observation is consistent with the concept that a larger lipid-rich necrotic core may increase plaque instability (22). Although direct pathological confirmation is lacking in our cohort, it is reasonable to speculate that increased NRS plaque burden may represent a systemic vulnerability state, which could predispose patients to progressive neurological deficits.

Several mechanisms may explain the observed association. First, plaque instability is not confined to a single vascular segment but often reflects a systemic inflammatory and pro-atherogenic milieu. Such a state may simultaneously affect extracranial and intracranial vessels, including perforating arteries (22). Second, carotid plaques with NRS often represent high-risk vulnerable lesions even with mild luminal narrowing. Plaques with irregular contours and necrotic cores can drastically alter local vascular shear stress, trigger microemboli shedding, and produce functional arterial stenosis despite limited luminal narrowing. Such pathological changes can reduce distal cerebral perfusion. In LSA-territory infarcts with inherently poor collateral circulation, additional perfusion insufficiency further increases the risk of progressive neurological deficits (23).

In addition, our study found that moderate-to-severe ICAS in non-culprit intracranial arteries was common in patients with END. Patients with moderate-to-severe ICAS exhibited a higher percentage of NRS plaques (13, 17). Stenosis in other arterial segments further suggests underlying atherosclerotic plaque and plaque instability, which may elevate the risk of perforating artery orifice obstruction (23). In terms of plaque area, no intergroup difference was observed, potentially due to the limited sample size and insufficient statistical power. Further studies are warranted to validate these results. Notably, the present study only demonstrates an independent association between carotid NRS plaque burden and END rather than a causal relationship. This association may reflect shared systemic inflammatory and atherosclerotic vulnerability across extracranial and intracranial vessels, rather than direct pathological causation.

END in BAD remains a clinical challenge, however existing antithrombotic treatment strategies lack strong evidence-based support (24). In clinical practice, intensified antithrombotic therapy is often initiated only after END onset, a point at which irreversible ischemic lesions and neurologic deficits may have already occurred (2, 25, 26). If carotid NRS plaque burden can help identify patients at higher risk of END at an early stage, it may provide additional information for risk stratification and closer monitoring. Whether such information should influence therapeutic decisions requires further prospective investigation. While causality cannot be established in this observational study, the results provide a pathophysiological framework linking extracranial plaque instability to perforator stroke progression.

This study has several limitations that should be acknowledged. First, as an observational study, residual confounding cannot be entirely excluded even after adjustment for multiple clinical variables. Unmeasured covariates may still affect the observed associations between carotid NRS plaques and END. Second, the retrospective single-centre design may lead to selection bias and reduce the representativeness of our cohort. Third, without an independent external validation cohort, our predictive results cannot be directly generalized to other stroke populations or clinical contexts. Finally, owing to its retrospective observational nature, causal links between carotid NRS plaques and END cannot be determined based on the present data. Future multicentre prospective cohorts with external validation are needed to verify our conclusions.

In conclusion, our study showed that an increased carotid NRS plaque burden is associated with END and ICAS in patients with BAD. Earlier detection of NRS plaques may help identify patients at higher risk of END and provide additional information for risk stratification.

## Data Availability

The original contributions presented in the study are included in the article/supplementary material, further inquiries can be directed to the corresponding author.
